# Neuropsychological profile in young girls at high risk of developing anorexia nervosa

**DOI:** 10.1002/erv.3151

**Published:** 2024-11-15

**Authors:** Karin Dahlin, Kajsa Järvholm, Sandra Rydberg Dobrescu, Jovanna Dahlgren, Elisabet Wentz

**Affiliations:** ^1^ Department of Psychiatry and Neurochemistry Institute of Neuroscience and Physiology University of Gothenburg Gothenburg Sweden; ^2^ Department of Psychology Faculty of Social Sciences Lund University Lund Sweden; ^3^ Gillberg Neuropsychiatry Centre Institute of Neuroscience and Physiology University of Gothenburg Gothenburg Sweden; ^4^ Region Västra Götaland Queen Silvia Children's Hospital Sahlgrenska University Hospital Gothenburg Sweden; ^5^ Department of Pediatrics Institute of Clinical Sciences, University of Gothenburg Gothenburg Sweden

**Keywords:** aetiology, anorexia nervosa, neuropsychology, prevention, risk factors

## Abstract

**Objective:**

Previous research has shown anorexia nervosa (AN) to be associated with a specific neuropsychological profile, including set‐shifting and central coherence deviances. A similar profile has been shown in adult unaffected relatives. The aim of this study was to examine whether poor set‐shifting and central coherence abilities could be detected in children at high risk of developing AN.

**Method:**

Twenty‐eight biological healthy daughters of women with previous or current AN and 42 biological daughters of healthy women, all between six and 12 years of age, participated in the study. A neuropsychological test battery (Wechsler Intelligence Scale for Children, Wisconsin Card Sorting Test, Trail Making Test and Rey Complex Figure Test) was used to assess set‐shifting and central coherence abilities.

**Results:**

No differences in set‐shifting or central coherence performance were detected between the high‐risk group and the comparison group. Adjustments for age and intelligence quotient (IQ) did not affect the results.

**Conclusions:**

Our results did not support the notion of preexisting neuropsychological deficits in AN‐related cognitive domains among high‐risk girls.

## Highlights

- No deviances in set‐shifting abilities were found among young girls at high risk of developing anorexia nervosa.
- No deviances in central coherence abilities were found among young girls at high risk of developing anorexia nervosa.
- The young girls at high risk of developing anorexia nervosa performed at par with a low‐risk comparison group regarding cognitive ability.

## 1 INTRODUCTION AND AIMS

Anorexia nervosa (AN) is a severe psychiatric disorder associated with an elevated mortality risk (van Eeden et al., 2021). AN is highly familial and the heritability estimated from twin studies is at least 50% (Sullivan et al., 2018; Yilmaz et al., 2015). The lifetime prevalence in females is up to 2% for strictly defined AN, and up to 4% when using a broader definition of the disease (Treasure et al., 2015; van Eeden et al., 2021). The female prevalence for AN is at least 10 times as high as for males. The incidence rate for AN has its peak in adolescence, with 15 years being the most common age of onset (van Eeden et al., 2021). Psychological treatment is still the most effective option in AN, although the outcome differs across the lifespan. For children and adolescents, Family‐Based Treatment is relatively effective whilst treatment for adults remains less successful (Treasure et al., 2015). Combining the age of onset with the immense psychological, physiological, and social impacts associated with AN, the importance of early detection and early treatment interventions becomes evident (Treasure et al., 2015).

### 1.1 Neuropsychological profile in anorexia nervosa

The large body of eating disorder (ED) research has mainly focused on the acute and recovered stages of AN, and essential knowledge about the intellectual and cognitive manifestations of ongoing AN has been gathered (Gillberg et al., 1996; Lopez et al., 2010; Smith et al., 2018). No significant differences in general intelligence quotient (IQ) have been found in either adolescents or adults, compared with healthy controls (Lang et al., 2015; Lopez et al., 2010; Lozano‐Serra et al., 2014; Weider et al., 2014). However, previous research has suggested the presence of a specific neuropsychological profile in individuals with AN. This ‘profile’ primarily consists of deficiencies in cognitive domains related to executive functioning and visuospatial processing, possibly abetting the AN psychopathology (Treasure et al., 2015). Specifically, deficiencies in set‐shifting (cognitive flexibility) and central coherence abilities have been considered possible candidates of the AN endophenotype (Holliday et al., 2005).

Set‐shifting refers to the ability to alternate focus between tasks or sets in accordance with situational demands. Deficiencies in set‐shifting have been suggested to explain core features in AN such as perseveration, rigidity, and obsessiveness (Holliday et al., 2005). Research into set‐shifting abilities and AN has generated mixed results. Poor set‐shifting in adolescents (Lang et al., 2015; Lozano‐Serra et al., 2014; Wu et al., 2014) and adults with AN has been reported in multiple studies (Holliday et al., 2005; Roberts et al., 2010; Stedal et al., 2021; Tchanturia et al., 2012; Wu et al., 2014). However, in children and adolescents, studies have also indicated a lack of impairment when comparing individuals with AN with healthy controls (Herbrich et al., 2018; Kjaersdam et al., 2015). Likewise, more recent reviews have shown less prominent deficiencies in adolescents compared with adults with AN (Lang, Stahl, et al., 2014; Stedal et al., 2021, 2022). In adults with AN, difficulties seem to persist after recovery, albeit to a lesser degree, supporting the notion that poor set‐shifting may be a preexisting cognitive trait, exacerbated by the onset of AN (Roberts et al., 2010; Talbot et al., 2015; Tchanturia et al., 2012).

Central coherence is the ability to integrate global information in order to apprehend the bigger picture. In AN, deviances in central coherence are suggested to explain common traits and behaviours such as perfectionism, obsessiveness (Lang, Lopez, et al., 2014), and the tendency to pay disproportionate attention to individual details such as body weight and shape (Smith et al., 2018). Weak central coherence has been found in both adolescents (Lang et al., 2015; Stedal et al., 2022) and adults with AN (Lang et al., 2014b, 2016a; Lopez et al., 2008; Stedal et al., 2021), which implies a more detail‐oriented processing style. However, findings regarding recovered AN are ambiguous (Fuglset, 2019), some studies supporting the notion of weak central coherence as independent of disease status (Tenconi et al., 2010), while others report no apparent differences in central coherence between individuals recovered from AN and healthy controls (Lang, Roberts, et al., 2016).

### 1.2 Premorbid neuropsychological functioning

Although the research is limited and ambiguous, there is evidence suggesting that set‐shifting and central coherence deviances are independent of disease status (Fuglset, 2019; Tenconi et al., 2010). To clarify this, a deeper understanding of the early or premorbid stages of AN is needed. Previously, the main research approach has been to acquire information on premorbid functioning retrospectively (Råstam, 1992; Råstam & Gillberg, 1992). A superior method is familial high‐risk (FHR) studies, which target individuals at high risk of developing a specific disease due to familial aggregation. Since the offspring of women with AN are up to 11 times more likely to develop the disease compared with the offspring of healthy controls, the former group constitutes a high‐risk sample for developing AN (Steinhausen et al., 2015). Combined with the elevated prevalence of AN in women, daughters of an affected mother run a particularly high risk.

FHR studies have scarcely been used in ED research, and the method is even less frequently applied for examining neuropsychological profiles in the offspring of mothers with a history of AN. Similarities in neuropsychological performance have previously been found between individuals with AN and their unaffected relatives, including mothers and adult sisters (Galimberti et al., 2013; Holliday et al., 2005; Lang et al., 2016a, 2016b; Tenconi et al., 2010). In addition, a large population‐based cohort study, the Avon Longitudinal Study of Parents and Children (ALSPAC), has examined children of mothers with ED at ages 8 and 10, reporting higher full‐scale and performance IQ, increased working memory capacity and decreased attentional control compared with children at low risk of developing an ED (Kothari et al., 2013). Further, specific neuropsychological difficulties (attention, working memory and inhibition) in childhood were identified as possible risk factors for symptoms of AN in adolescence (Schaumberg et al., 2020). To date, there is only one published FHR study including daughters (8–15 years old) of women with a history of AN or bulimia nervosa (BN). In addition to a cognitive assessment, the children were examined using magnetic resonance imaging (MRI) of the brain regarding functional connectivity in resting‐state networks. Preliminary findings showed poorer set‐shifting abilities and a possible trend towards increased working memory performance in the sample consisting of 16 high‐risk daughters compared with 20 control girls. The researchers suggested that the results implicate an ED endophenotype (Pappaianni et al., 2023).

### 1.3 Purpose and aims

Utilising the FHR method, the aim of this study was to examine neuropsychological functioning in daughters of mothers with previous or current AN compared with daughters of unafflicted mothers. We hypothesised that the neuropsychological deficits regarding set‐shifting and central coherence seen in individuals with AN could also be detected in girls at high risk of developing AN, and that these girls would perform worse on tasks measuring set‐shifting and central coherence compared with a matched group of girls at low risk of developing AN.

## 2 METHOD

### 2.1 Participants

#### 2.1.1 The high‐risk group

The following criteria were used for inclusion of the study participants: biological daughter of mother with previous or current AN, and between six and 12 years of age. The age range was chosen to minimise the probability of pubertal onset, thus avoiding the risk of including girls with an already debuted AN. More than one daughter per mother were allowed to participate in the study. The only exclusion criterion was a previous or current AN diagnosis. In total, 28 girls were recruited to the high‐risk group, including six pairs of siblings. The participants were approached (a) through their mothers, at the ED services throughout Region Västra Götaland (a county in the southwest of Sweden with a population of 1,750,000) (*N* = 22), and (b) through the Swedish association ‘Frisk & Fri’ (“Healthy & Free”), an organisation for individuals with a history of EDs and their relatives (*N* = 6).

#### 2.1.2 The comparison group

An age‐matched comparison group was recruited and included 42 daughters whose mothers had no history of ED. The comparison group was recruited through a variety of sources, including advertisements in local newspapers in Gothenburg and surrounding municipalities in Sweden. Bill‐posting in public spaces and digital advertisements on the external website of the Region Västra Götaland were also used. Finally, employees from two clinics at Sahlgrenska University hospital in Gothenburg were informed about the study via e‐mail. One pair of siblings was included in the comparison group.

### 2.2 Procedure

The present study was part of an overarching research project. Therefore, the examination procedure included various elements of data collection, in addition to the neuropsychological testing presented in this article. The examinations took place at the research department at Queen Silvia children's hospital/Sahlgrenska University hospital in Gothenburg and were scheduled in the morning. Blood samples were collected fasting and after breakfast. The participant's height and weight were measured and growth charts with previous growth data were collected. The neuropsychological tests were then administered. The examination took approximately 3 hours. For three of the high‐risk group participants, examinations were moved to a location closer to their homes. In these cases, blood samples could not be collected.

Meanwhile, to verify a previous or current diagnosis of AN, the mothers in the high‐risk group were interviewed by a psychiatrist (last author, EW) using the ED module of the Structured Clinical Interview for DSM‐IV (SCID‐I) (First et al., 1997). A supplementary checklist of criteria for EDs based on the Diagnostic and Statistical Manual of Mental Disorders (DSM‐5) (American Psychiatric Association, 2013) was added to examine the occurrence of a previous or current AN diagnosis according to the DSM‐5. Information on the duration of the ED was also collected. In addition, the interviews were used to rule out any ED occurrence in the mothers of the comparison group. Demographic data regarding the parents' level of education and employment status were collected. Subsequently, parental questionnaires were administered to screen for psychiatric symptoms among the participants. To assess behavioural problems, the parental version of the Strengths and Difficulties Questionnaire (SDQ) (Goodman, 1997) was used. The SDQ generates a total difficulties score, range 0–40. In the validated Swedish version of the SDQ, a cut‐off level at ≥14 has been suggested (Malmberg et al., 2003). The participant and the mother were also asked to participate in a structural and functional magnetic resonance imaging (MRI) scan of the brain on a separate occasion. Results regarding blood samples, nutritional data, psychiatric morbidity and brain imaging will be presented in subsequent publications.

The data collection started in October 2020 and was mainly carried out during the COVID‐19 pandemic, which to some extent affected the examination procedures and the total period needed to collect the data. During the pandemic, all examinations had to adhere to the current restrictions and were cancelled and rescheduled if the participant or their mother reported any symptoms of infection. Since the sample groups consisted of healthy individuals, permission had to be given by the hospital management group before the study examinations began. Data collection ended in June 2023.

### 2.3 Neuropsychological tests

**FIGURE 1:**
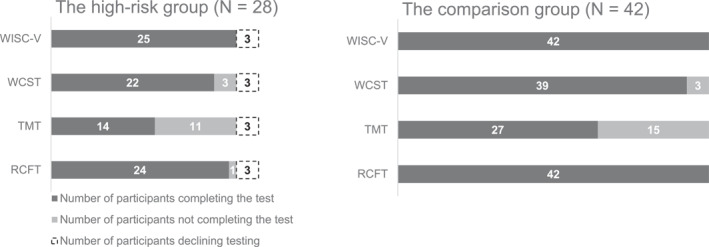
Neuropsychological test battery and number of participants completing the tests in the high‐risk group and the comparison group. The left bar chart displays the high‐risk group and the right bar chart shows the comparison group. Each bar in the charts represents the number of participants completing, not completing and declining participation for each of the test instruments included. Due to the age restrictions of the TMT, the test was only administered to participants >8 years of age. RCFT, Rey complex figure test; TMT, Trail making test; WCST, Wisconsin card sorting test; WISC‐V, Wechsler intelligence scale for children—fifth edition.

The neuropsychological test battery was administered to 25 of 28 participants in the high‐risk group and to all 42 participants in the comparison group. Figure 1 provides an overview of the number of participants completing each test. The test instruments were administered by the first author (KD), who is a licenced psychologist.

#### 2.3.1 Wechsler intelligence scale for children—Fifth edition (WISC‐V)

General intelligence and cognitive abilities were assessed through digital administration of the Swedish version of the Wechsler Intelligence Scale for Children—Fifth Edition (WISC‐V) (Wechsler, 2016). The WISC‐V is a well‐known and widely used psychological test for children. All the primary subtests were administered to generate composite scores for both general intelligence and functioning in specific cognitive domains. The Full Scale Intelligence Quotient (FSIQ) score, representing the overall intellectual ability, and the Primary Index Scores were calculated. The Primary Index Scores include the following cognitive domains; Verbal Comprehension Index (VCI), Visual Spatial Index (VSI), Fluid Reasoning Index (FRI), Working Memory Index (WMI), and Processing Speed Index (PSI). The General Abilities Index (GAI), comprising three subtests of the VCI index and three subtests from the VSI index, was also calculated. Compared with the FSIQ, the GAI delivers an estimate of general intelligence that is less affected by working memory and processing speed. Of the 25 participants in the high‐risk group who agreed to undergo neuropsychological testing, 24 completed the WISC‐V during the study examination. One high‐risk group participant had completed the WISC‐V 3 weeks earlier, and her WISC‐V results were obtained from the clinic where she was tested. All participants in the comparison group (*N* = 42) completed the WISC‐V. Due to technical difficulties, two of the comparison group participants were administered the paper‐and‐pencil version of the WISC‐V.

#### 2.3.2 Wisconsin card sorting test

The Wisconsin Card Sorting Test (WCST) is a well‐established instrument for assessing set‐shifting abilities in participants aged 6–89 years. The WCST requires the participant to sort cards according to an unknown sorting rule, thus providing information about the response strategies used (Heaton et al., 1993). In research, a selection of WCST sub‐scores is commonly reported. In the present study, the WCST Computer Version 4 (Wisconsin Card Sorting Test, 2008) was administered. To enable comparisons across ages, the normalised standard scores (*M* = 100) were used for the number of perseverative errors, number of total errors and number of perseverative responses. A higher number indicates a better performance. The number of completed categories was also calculated (0–6). Number of perseverative errors functioned as the primary outcome variable. In the high‐risk group (*N* = 25), three participants were not able to complete the WCST (two declined due to fatigue, one was unable to complete the task owing to a technical error). In the comparison group (*N* = 42), three participants were unable to complete the task due to a technical error.

#### 2.3.3 Trail making test

The Trail Making Test (TMT) is a subtest in the Delis‐Kaplan Executive Function System (Delis et al., 2001). The test measures set‐shifting through a set of five visuo‐motor tasks and is suitable for individuals aged ≥8 years. In the Swedish version of the TMT, the supplement for American normative scores is used. In this study, scaled scores for all five TMT tasks were calculated. However, the task of primary interest regarding set‐shifting is Condition 4: the number‐letter switching condition. The condition requires the test person to draw a line, alternating between numbers (numerical) and letters (alphabetical). Due to the age restrictions of the TMT, the test was not administered to participants <8 years of age. In total, 14 of the participants in the high‐risk group and 27 in the comparison group were assessed with the TMT. One comparison group participant was unable to complete Condition 4 because she had yet to learn the alphabet.

#### 2.3.4 Rey complex figure test

The Rey Complex Figure Test (RCFT) (Osterrieth, 1944) is a widely used neuropsychological test, which captures various cognitive abilities. In this study, the copy condition of the RCFT was used to assess central coherence. The condition requires the test person to copy a complex figure onto a blank sheet of paper. Initially, the accuracy of the copy task was calculated using the original scoring principals (Meyers & Meyers, 1995). Subsequently, two alternative scoring indices were applied to measure central coherence ability. The first index, the Booth scoring system, is a well‐established method for measuring central coherence in ED research (Booth, 2006; Harrison et al., 2011; Herbrich et al., 2018; Lang et al., 2015; Lopez et al., 2008; Rose et al., 2014), incorporating the constructural organisation of the figure (OCI; order of construction index, mean range 0–3.3) as well as the drawing style (SI; style index, mean range 0–2), ultimately generating a central coherence index (CCI), mean range 0–2. In this study, the original Booth scoring system, as described by Lopez (Lopez et al., 2008), was applied. The CCI was calculated by summing the proportion of total possible scores for OCI (score/3.3) and SI (score/2).

Since the study sample consisted of children, an additional index specifically designed to account for developmental variabilities in childhood was also calculated; the Rey Complex Figure Organizational Strategy Score (RCF‐OSS). Although less established, the RCF‐OSS has acceptable inter‐rater reliability and temporal stability. The system was developed to assess the organizational strategies used at the start and in the process of drawing the figure. The RCF‐OSS ultimately generates a combined score on a seven‐point scale, level 1 indicating a lack of organisation and level 7 representing the highest organisation. In between are levels of poor (level 2), random (level 3), fragmented (level 4), part‐configural (level 5) and conceptual (level 6) organisation (Anderson et al., 2001). Since the RCF‐OSS is a less recognized and less frequently used central coherence index, 10% of the RCF‐OSS assessments were independently rated by two raters in the study to assess inter‐rater agreement. To ensure equal representation across the age span, 50% of the inter‐ratings were selected from the youngest and eldest half of the sample, respectively. The inter‐rater agreement was ‘very good’, with Cohen's kappa = 1.00.

### 2.4 Statistical analysis

A linear mixed effect model was used for analyses of the primary and secondary outcome measures, comparing the high‐risk group and the comparison group. The model was applied to account for non‐independence within the data set, that is, clustering of siblings and correlations between daughters of the same mother. Study group, age, and FSIQ were included as fixed effects and mother as random effect. Robust standard errors (HC3 method) were used to account for possible violations against distributional assumptions. The standardized effect size, calculated as the mean difference or adjusted mean difference divided by the pooled standard deviation, was calculated. Multiple imputation was applied in the analyses to handle missing data, using regression imputation for numeric variables and logistic regression imputation for ordinal variables. In line with the applied model above, the study group, age and FSIQ were used as auxiliary variables for the imputation as these were assumed to predict the missing data and for compatibility between imputation model and analysis model. All analyses were performed both unadjusted and adjusted for age, since age‐matched sample groups imply adjustment for age by design (Bland & Altman, 1994). Additionally, we adjusted for the FSIQ in order to estimate the difference in cognitive function for the same level of IQ. No further adjustments were made since these were either deemed superfluous after accounting for the child's IQ (e.g., maternal income and educational level) or were considered as mediators rather than confounders (e.g., the daughter's characteristics).

All tests were two‐tailed and conducted at the 5% significance level. Statistical analyses were performed using the SAS/STAT^®^ Software, Version 9.4 of the SAS System for Windows (SAS Institute Inc.).

### 2.5 Ethics approval

The study was approved by the Swedish Ethical Review Authority on 7 February 2020 (2019–05669). Written informed consent was obtained from all the caregivers and assent was collected from the participants.

## 3 RESULTS

A detailed description of the demographic characteristics of the participants and their biological mothers is displayed in Table 1.

**TABLE 1:** Demographic characteristics of participants and participants' biological mothers.

Participants	High‐risk group	Comparison group
	*N* = 28^a^	*N* = 42^b^
	Mean (SD)	Mean (SD)
Age at examination (years)	8.9 (2.3)	8.9 (1.9)
Age BMI SDS (years)	8.9 (2.2)	8.9 (1.9)
Weight (kilogramme)	33.2 (11.9)	30.1 (7.4)
Height (centimetre)	135.7 (15.2)	134.7 (13.2)
BMI SDS^c^	0.4 (1.4)	−0.1 (1.1)

	*N* (%)	*N* (%)
Pubertal stage (tanner)		
Stage 1	19 (67.9)	27 (64.3)
Stage 2	5 (17.9)	11 (26.2)
Stage 3	3 (10.7)	4 (9.5)
Stage 4	1 (3.6)	0 (0.0)
Menarche	2 (7.1)	0 (0.0)

	Mean (SD)	Mean (SD)
WISC‐V^d^		
FSIQ	98.2 (11.0)	107.4 (13.0)
GAI	99.6 (10.8)	108.7 (12.3)
VCI	101.7 (15.1)	109.9 (13.9)
VSI	97.3 (12.9)	106.4 (10.8)
FRI	97.4 (10.5)	105.5 (12.6)
WMI	95.3 (11.5)	103.2 (15.0)
PSI	96.3 (9.4)	101.1 (13.4)
SDQ total difficulties score	7.8 (6.5)	5.8 (5.3)

Biological mothers	*N* = 22	*N* = 41
	Mean (SD)	Mean (SD)
Age (years)	41.2 (6.5)	42.1 (4.5)
Duration of first AN period (years)	9.8 (9.3)	n.a.
Duration of all AN periods (years)	12.7 (10.8)	n.a.
Duration of all ED periods	15.5 (10.1)	n.a.

	*N* (%)	*N* (%)
Current AN	12 (54.5)	n.a.
University degree	14 (63.6)	36 (87.8)
Employment past 6 months		
No employment^e^	10 (45.5)	2 (4.9)
Part‐time employment^f^	4 (18.2)	2 (4.9)
Full‐time employment^g^	8 (36.4)	37 (90.2)

*Note*: Data are presented as means and standard deviations for numeric variables and as numbers and percentages for categorical variables.

Abbreviations: AN, Anorexia nervosa; BMI SDS, Body mass index standard deviation score; ED, Eating disorder; FRI, Fluid reasoning index; FSIQ, Full scale intelligence quotient; GAI, General abilities index; n.a., not applicable; PSI, Processing speed index; SDQ, Strengths and difficulties questionnaire; VCI, Verbal comprehension index; VSI, Visual spatial index; WISC‐V, Wechsler intelligence scale for children‐fifth edition; WMI, Working memory index.

^a^ The sample includes six pairs of siblings.

^b^ The sample includes one pair of siblings.

^c^ BMI SDS was based on age, weight and height at examination. For two high‐risk group participants, BMI SDS calculations were based on an examination performed approximately 1 year earlier. One high‐risk group participant declined to have her weight measured; calculations were therefore based on the mother's estimate of the participant's weight and height.

^d^ WISC‐V results were obtained for all participants except three individuals in the high‐risk group who declined neuropsychological testing.

^e^ No employment: Unemployed, sick leave, sickness benefit.

^f^ Part‐time employment: Refers to mothers whose occupation varied between ‘No employment’ and ‘Full‐time employment’ during the time period in question.

^g^ Full‐time employment: Employed, student and/or parental leave.

### 3.1 Neuropsychological tests

Table 2 presents results from the neuropsychological tests for the high‐risk group and comparison group, respectively. The results were first analysed without adjustment, and thereafter with adjustments for ‘age’ and ‘age plus FSIQ’ (Table 2). Table 3 shows the results after using multiple imputation for missing data.

**TABLE 2:** Primary and secondary analyses of cognitive function in the high‐risk group versus comparison group with available data.

Variable	High‐risk group (*N* = 28)	Comparison group (*N* = 42)	Mean difference (95% CI)		
			Unadjusted	Adjusted 1^a^	Adjusted 2^b^
Wisconsin card sorting test					
No. perseverative errors	102.2 (11.1) *N* = 22	104.7 (15.8) *N* = 39	−2.50 (−9.21 to 4.20) *p* = 0.46	−1.84 (−7.98 to 4.31) *p* = 0.55	−1.55 (−8.16 to 5.05) *p* = 0.64
No. total errors	101.4 (14.1) *N* = 22	102.7 (17.9) *N* = 39	−1.31 (−8.91 to 6.29) *p* = 0.73	−0.56 (−7.65 to 6.53) *p* = 0.88	1.10 (−6.67 to 8.86) *p* = 0.78
No. perseverative responses	101.4 (11.1) *N* = 22	104.8 (14.9) *N* = 39	−3.33 (−9.86 to 3.20) *p* = 0.31	−2.66 (−8.59 to 3.26) *p* = 0.37	−2.36 (−8.79 to 4.08) *p* = 0.47
No. completed categories	5.14 (1.49) *N* = 22	4.53 (1.90) *N* = 39	0.61 (−0.29 to 1.50) *p* = 0.18	0.68 (−0.13 to 1.49) *p* = 0.097	0.55 (−0.39 to 1.49) *p* = 0.24
Rey complex figure test					
Total accuracy score	25.3 (8.0) *N* = 24	26.8 (7.2) *N* = 42	−1.45 (−5.52 to 2.62) *p* = 0.48	−1.09 (−3.92 to 1.74) *p* = 0.44	0.38 (−2.50 to 3.25) *p* = 0.79
RCF‐OSS	4.02 (1.30) *N* = 24	4.26 (1.17) *N* = 42	−0.24 (−0.94 to 0.46) *p* = 0.49	−0.24 (−0.82 to 0.34) *p* = 0.41	−0.25 (−0.81 to 0.31) *p* = 0.37
Booth CCI	1.37 (0.16) *N* = 24	1.35 (0.24) *N* = 42	0.02 (−0.09 to 0.13) *p* = 0.71	0.02 (−0.08 to 0.12) *p* = 0.70	0.01 (−0.10 to 0.11) *p* = 0.92
Booth OCI	2.16 (0.32) *N* = 24	2.15 (0.36) *N* = 42	0.01 (−0.18 to 0.20) *p* = 0.95	0.00 (−0.18 to 0.19) *p* = 0.97	−0.06 (−0.24 to 0.13) *p* = 0.53
Booth SI	1.42 (0.29) *N* = 24	1.39 (0.35) *N* = 42	0.03 (−0.14 to 0.21) *p* = 0.70	0.03 (−0.14 to 0.21) *p* = 0.69	0.05 (−0.14 to 0.23) *p* = 0.63
Trail making test^c^					
Condition 1 visual scanning	8.35 (3.60) *N* = 14	9.15 (3.21) *N* = 27	−0.80 (−3.26 to 1.66) *p* = 0.51	−0.92 (−3.49 to 1.64) *p* = 0.47	−0.54 (−3.28 to 2.20) *p* = 0.69
Condition 2 number sequencing	8.96 (3.53) *N* = 14	10.6 (3.0) *N* = 27	−1.62 (−4.08 to 0.85) *p* = 0.19	−1.68 (−4.20 to 0.83) *p* = 0.18	−0.69 (−3.04 to 1.65) *p* = 0.55
Condition 3 letter sequencing	9.00 (3.04) *N* = 14	10.00 (4.00) *N* = 27	−1.00 (−3.18 to 1.18) *p* = 0.36	−1.14 (−3.44 to 1.15) *p* = 0.32	0.47 (−1.71 to 2.65) *p* = 0.66
Condition 4 number‐letter switching	8.50 (4.36) *N* = 14	9.88 (3.53) *N* = 26	−1.38 (−4.11 to 1.34) *p* = 0.31	−1.61 (−4.39 to 1.18) *p* = 0.25	−0.09 (−3.14 to 2.97) *p* = 0.96
Condition 5 motor speed	9.89 (3.26) *N* = 14	10.2 (3.1) *N* = 26	−0.29 (−2.65 to 2.06) *p* = 0.80	−0.20 (−2.62 to 2.22) *p* = 0.87	−0.24 (−3.03 to 2.55) *p* = 0.86

*Note*: Descriptive data are presented as mean and standard deviation with the number of row available. Comparisons between groups were performed using linear mixed effect models with mother as random effect to account for clustering of siblings and correlations between daughters to the same mother. Robust standard errors (HC3 method) were used to account for possible violations against distributional assumptions.

Abbreviations: CCI, Central coherence index; CI, confidence interval; FSIQ, Full scale intelligence quotient; OCI, Order of construction index; RCF‐OSS, Rey complex figure ‐ organizational strategy score; SI, style index.

^a^ Adjusted for child's age.

^b^ Additionally adjusted for FSIQ.

^c^ Only administered to participants aged 8–12 years. *N* = 14 in the high‐risk group and 27 in the comparison group.

**TABLE 3:** Primary and secondary analyses of cognitive function in the high‐risk group versus comparison group using multiple imputation to account for missing data.

Variable	High‐risk group (*N* = 28)	Comparison group (*N* = 42)	Mean difference (95% CI)		
			Unadjusted	Adjusted 1^a^	Adjusted 2^b^
Wisconsin card sorting test					
No. perseverative errors	102.3 (12.4)	104.3 (15.6)	−2.04 (−9.63 to 5.55) *p* = 0.59	−2.05 (−9.18 to 5.08) *p* = 0.57	−1.66 (−8.91 to 5.60) *p* = 0.65
No. total errors	101.4 (15.3)	102.2 (17.8)	−0.80 (−9.57 to 7.97) *p* = 0.86	−0.81 (−9.05 to 7.43) *p* = 0.84	0.99 (−7.17 to 9.14) *p* = 0.81
No. perseverative responses	101.6 (12.2)	104.5 (14.8)	−2.86 (−10.18 to 4.46) *p* = 0.44	−2.87 (−9.71 to 3.97) *p* = 0.40	−2.44 (−9.45 to 4.58) *p* = 0.49
No. completed categories	5.15 (1.49)	4.54 (1.89)	0.62 (−0.29 to 1.52) *p* = 0.18	0.61 (−0.24 to 1.46) *p* = 0.16	0.52 (−0.42 to 1.45) *p* = 0.27
Rey complex figure test					
Total accuracy score	25.8 (8.0)	26.8 (7.2)	−0.97 (−5.04 to 3.10) *p* = 0.63	−1.06 (−3.93 to 1.82) *p* = 0.47	0.42 (−2.34 to 3.18) *p* = 0.76
RCF‐OSS	4.04 (1.31)	4.26 (1.17)	−0.22 (−0.91 to 0.48) *p* = 0.54	−0.24 (−0.84 to 0.36) *p* = 0.42	−0.23 (−0.84 to 0.37) *p* = 0.45
Booth CCI	1.37 (0.17)	1.35 (0.24)	0.02 (−0.09 to 0.14) *p* = 0.68	0.02 (−0.09 to 0.13) *p* = 0.69	0.00 (−0.11 to 0.12) *p* = 0.96
Booth OCI	2.16 (0.33)	2.15 (0.36)	0.01 (−0.19 to 0.20) *p* = 0.93	0.00 (−0.18 to 0.19) *p* = 0.96	−0.05 (−0.24 to 0.15) *p* = 0.63
Booth SI	1.43 (0.30)	1.39 (0.35)	0.04 (−0.14 to 0.22) *p* = 0.65	0.04 (−0.14 to 0.22) *p* = 0.66	0.03 (−0.16 to 0.23) *p* = 0.72
Trail making test^c^					
Condition 1 visual scanning	8.31 (3.58)	9.16 (3.21)	−0.85 (−3.23 to 1.53) *p* = 0.48	−1.02 (−3.46 to 1.43) *p* = 0.41	−0.54 (−3.16 to 2.08) *p* = 0.68
Condition 2 number sequencing	8.94 (3.49)	10.6 (3.0)	−1.65 (−3.97 to 0.67) *p* = 0.16	−1.69 (−3.98 to 0.60) *p* = 0.14	−0.93 (−3.25 to 1.38) *p* = 0.42
Condition 3 letter sequencing	8.97 (3.18)	10.00 (4.00)	−1.03 (−3.35 to 1.30) *p* = 0.38	−1.22 (−3.56 to 1.13) *p* = 0.30	0.43 (−1.65 to 2.50) *p* = 0.68
Condition 4 number‐letter switching	8.59 (4.30)	9.63 (3.81)	−1.04 (−3.77 to 1.68) *p* = 0.45	−1.45 (−4.17 to 1.28) *p* = 0.29	−0.06 (−3.02 to 2.89) *p* = 0.97
Condition 5 motor speed	10.0 (3.2)	10.2 (3.1)	−0.16 (−2.38 to 2.06) *p* = 0.89	−0.21 (−2.44 to 2.02) *p* = 0.85	−0.09 (−2.72 to 2.53) *p* = 0.94

*Note*: Descriptive data are presented as mean and standard deviation. Comparisons between groups were performed using linear mixed effect models with mother as random effect to account for clustering of siblings and correlations between daughters to the same mother. Robust standard errors (HC3 method) were used to account for possible violations against distributional assumptions. Missing data were handled using multiple imputation.

Abbreviations: CCI, Central coherence index; CI, confidence interval; FSIQ, Full scale intelligence quotient; OCI, Order of construction index; RCF‐OSS, Rey complex figure ‐ organizational strategy score; SI, Style index.

^a^ Adjusted for child's age.

^b^ Additionally adjusted for FSIQ.

^c^ Only administered to participants aged 8–12 years. *N* = 14 in the high‐risk group and 27 in the comparison group. After imputation, *N* = 17 in the high‐risk group and 27 in the comparison group.

#### 3.1.1 Set‐shifting abilities

The primary outcome variable, the WCST number of perseverative errors, showed no significant differences between the groups. Equal results were also shown for the remaining WCST variables (number of total errors, perseverative responses and completed categories) (Table 2). Regarding the TMT, performed by participants between 8 and 12 years of age, analysis of scaled scores for TMT Condition 4 (number‐letter switching) revealed no significant group differences (Table 2). Similar results were found for the remaining TMT conditions. The use of multiple imputations and adjustments for age and the FSIQ, did not affect any of the set‐shifting variables (Table 3). A small to moderate effect size was found for TMT Condition 2 (number sequencing) using available data (Table 4). All other set‐shifting effect sizes were low.

**TABLE 4:** Primary and secondary analyses of cognitive function in the high‐risk group versus comparison group with available data; standardized effect size.

Variable	Standardized effect size (95% CI)		
	Unadjusted	Adjusted 1^a^	Adjusted 2^b^
Wisconsin card sorting test			
No. perseverative errors	−0.18 (−0.64 to 0.29)	−0.13 (−0.56 to 0.30)	−0.11 (−0.57 to 0.35)
No. total errors	−0.08 (−0.53 to 0.38)	−0.03 (−0.46 to 0.39)	0.07 (−0.40 to 0.53)
No. perseverative responses	−0.24 (−0.72 to 0.23)	−0.19 (−0.63 to 0.24)	−0.17 (−0.64 to 0.30)
No. completed categories	0.34 (−0.16 to 0.85)	0.38 (−0.07 to 0.84)	0.31 (−0.22 to 0.84)
Rey complex figure test			
Total accuracy score	−0.19 (−0.74 to 0.35)	−0.15 (−0.52 to 0.23)	0.05 (−0.33 to 0.43)
RCF‐OSS	−0.20 (−0.77 to 0.37)	−0.20 (−0.67 to 0.28)	−0.20 (−0.66 to 0.25)
Booth CCI	0.09 (−0.41 to 0.59)	0.09 (−0.38 to 0.56)	0.03 (−0.47 to 0.52)
Booth OCI	0.02 (−0.52 to 0.56)	0.01 (−0.51 to 0.53)	−0.16 (−0.69 to 0.36)
Booth SI	0.10 (−0.43 to 0.63)	0.10 (−0.42 to 0.62)	0.13 (−0.41 to 0.68)
Trail making test^c^			
Condition 1 visual scanning	−0.24 (−0.97 to 0.49)	−0.27 (−1.03 to 0.49)	−0.16 (−0.97 to 0.65)
Condition 2 number sequencing	−0.50 (−1.26 to 0.26)	−0.52 (−1.29 to 0.25)	−0.21 (−0.93 to 0.51)
Condition 3 letter sequencing	−0.27 (−0.86 to 0.32)	−0.31 (−0.93 to 0.31)	0.13 (−0.46 to 0.72)
Condition 4 number‐letter switching	−0.36 (−1.07 to 0.35)	−0.42 (−1.15 to 0.31)	−0.02 (−0.82 to 0.78)
Condition 5 motor speed	−0.09 (−0.82 to 0.64)	−0.06 (−0.82 to 0.69)	−0.07 (−0.94 to 0.79)

*Note*: The standardized effect size was calculated as the mean difference or adjusted mean difference divided by the pooled standard deviation (square root of residual variance plus variance of random intercept), accounting for correlations within families. The standardized effect size was interpreted according to Cohen's d; 0.2 = small, 0.5 = moderate and 0.8 = large.

Abbreviations: CCI, Central coherence index; CI, confidence interval; FSIQ, Full scale intelligence quotient; OCI, Order of construction index; RCF‐OSS, Rey complex figure ‐ organizational strategy score; SI, Style index.

^a^ Adjusted for child's age.

^b^ Additionally adjusted for FSIQ.

^c^ Only administered to participants aged 8–12 years. *N* = 14 in the high‐risk group and 27 in the comparison group.

#### 3.1.2 Central coherence abilities

Regarding the RCFT Total accuracy score and the two central coherence indices (RCF‐OSS and Booth CCI), the results showed equal performance between the two groups (Table 2). Imputations or other adjustments did not alter the results significantly (Table 3). All effect sizes for central coherence variables were low (Table 4).

## 4 DISCUSSION

The aim of the present study was to explore whether specific neuropsychological deviances associated with AN could be detected in children at high risk of developing the disease. In addition, we sought to compare the neuropsychological functioning of these high‐risk individuals with that of their low‐risk counterparts. In summary, our results did not support the notion of preexisting set‐shifting and central coherence deficits in high‐risk children. We found no significant differences regarding any of the outcome variables between the groups.

In this study, set‐shifting abilities were measured by two separate instruments: the WCST and the TMT. The variables of principal interest regarding set‐shifting, including the primary outcome ‘number of perseverative errors’, did not prove to be deviant among the high‐risk group individuals, showing results at par with the comparison group. Further, the analysis of TMT Condition 4 mirrored the WCST findings. The use of scaled and standardized scores for the WCST and the TMT allowed for comparisons across age groups, and the results for the set‐shifting performance on the tests indicated normal group level performances for both the high‐risk and the comparison group (Delis et al., 2001; Heaton et al., 1993). However, due to the TMT age restrictions, only a sub‐set of the sample completed this test.

Regarding central coherence, the RCFT was used to calculate two different central coherence indices. The Booth CCI, assessing organizational strategy and drawing style, has been widely used in adult and adolescent AN research (Booth, 2006; Harrison et al., 2011; Herbrich et al., 2018; Lang et al., 2015; Lopez et al., 2008; Rose et al., 2014). However, the use of the Booth method in younger children is limited (Rose et al., 2014). Since our sample consisted of children at an age where extensive cognitive development occurs, we used an additional index to enable a more reliable assessment of central coherence. The RCF‐OSS is specifically suitable for evaluating organizational strategy in early childhood, although the method has never been applied in ED research before. The analysis of both indices showed the same results; that is, no significant differences in central coherence abilities between children at high risk of AN and the comparison group. Thus, our hypothesis regarding central coherence deficiencies was not supported.

The apparent interpretation of our findings would be that neither set‐shifting deficiencies nor weak central coherence constitute premorbid traits of an AN pathology. This conclusion would indicate neuropsychological deviances to be disease‐dependent consequences or ‘scars’ of the starvation and malnourishment during the acute stages of AN. Further, it would be in contrast with the results of the preliminary FHR study published last year, which reported set‐shifting deficiencies in daughters of mothers with either AN or bulimia nervosa, using the Attention Switching Task of the computerised Cambridge Neuropsychological Test Automated Battery (CANTAB) (Pappaianni et al., 2023). Although methodologically similar, there are a few essential differences between the studies that presumably affect the results. Firstly, our sample consisted exclusively of the offspring of mothers with AN. The contrasting FHR study used a mixed sample; that is, daughters of mothers with either AN or BN. Secondly, different neuropsychological instruments were used across the two studies. Thirdly, the mean ages of the study sample groups were different. Although partly overlapping, our high‐risk group was considerably younger, thus limiting the risk of AN onset, while the other FHR study included girls closer to the peak age of AN onset.

To our knowledge, this is the first study utilising the WCST, TMT and RCFT on a sample of children with a high risk of developing AN. Therefore, multiple factors are central to interpreting the current outcome. Our results could not confirm the previous findings of first‐degree relatives (mothers and sisters) of individuals with AN, where similar neuropsychological profiles have been found in both affected and unaffected family members (Galimberti et al., 2013; Holliday et al., 2005; Lang et al., 2016a, 2016b; Tenconi et al., 2010). However, previous research has almost exclusively focused on adult relatives of individuals with AN. Further, studies conducted on adolescents in the acute stages of AN have shown less prominent deficiencies in set‐shifting and central coherence compared with their adult counterparts. Likewise, a meta‐analysis focusing on neuropsychological functioning in younger children with AN revealed a general non‐significant difference between subjects and controls. The exception was central coherence, memory and working memory, where significantly poorer results were seen in the children with AN. However, the need to interpret the results with caution was pointed out, due to the systematic use of test instruments originally developed for adults (Stedal et al., 2022).

In summary, the less evident neuropsychological deviances in younger individuals with AN imply that age and/or the duration of the illness mediate the extent of cognitive deficiencies in ongoing AN (Lang et al., 2015; Stedal et al., 2021, 2022). Nevertheless, the similarities found between adult individuals with AN and their relatives are indicative of premorbid cognitive deficits in AN. A possible explanation for these contradictory findings could be found in the cognitive development during childhood. In one study, the cognitive performance in children (9–14 years) and adolescents with AN were compared with the performance of healthy control children and adolescents. Although no significant differences were detected between children/adolescents with AN and control children/adolescents, exploratory analyses revealed that age‐related improvements in set‐shifting abilities were less pronounced in the AN sample compared with the control groups, suggesting a deviant *developmental trajectory* of set‐shifting in AN with the onset in childhood and adolescence, respectively (van Noort et al., 2016).

A prerequisite for defining a specific function as impaired is adequate knowledge about the typical development of that ability. Regarding the frontal lobe, including abilities of set‐shifting and central coherence, normal development is described as a multi‐stage process. Consequently, different cognitive abilities develop and mature in different ways and at different times. In general, the developmental trajectory is at its peak during age of 5–8 years, while moderate developmental processes continue to mature throughout childhood (Korkman et al., 2001). Regarding set‐shifting, a meta‐analysis has shown that the greatest age‐related increase in functioning occurs between 5‐8 years and 8–11 years of age, respectively. A continued, albeit smaller, increase occurs between 11 and 14 years of age (Romine & Reynolds, 2005). Further, research on typical development for central coherence is still lacking, but it has been suggested that the ability is really a form of processing style rather than an impairment or a skill. Looking into developmental aspects of central coherence derived from the RCFT specifically, studies have shown that although younger children may be aware of the larger constructural units of the Rey figure at an early age, this knowledge is not necessarily put to use when organising the task until age nine or later (Akshoomoff & Stiles, 1995). Considering the cognitive maturation during childhood, the demand for developmentally sensitive neuropsychological tests becomes evident. Notwithstanding, most instruments used for research purposes rely on the assumption that the test subject is a fully developed adult individual (Stedal et al., 2022).

Taken together, four likely interpretations could be made on the basis of our results. First, the lack of differences between our groups might indicate that cognitive deficiencies in set‐shifting and central coherence are simply not present in children at a high risk of developing AN, and thus not part of the suggested AN endophenotype; second, the relatively small sample size could have resulted in difficulties to detect significant group differences; third, our results could merely be a consequence of using test instruments that are inadequately tailored for measuring cognitive functions in young children, although well‐established and commonly used both in ED research and in clinical settings, and fourth, the high‐risk group might still present with neuropsychological deviances, although characterised as subtle deviances in the actual trajectory of cognitive development. The latter would correspond to previous findings of the acute stage of AN, showing more prominent cognitive deficiencies in older individuals. However, to ascertain whether the development of set‐shifting and central coherence is indeed deviant in our high‐risk individuals, a follow‐up study would be needed. To our knowledge, there are no previous longitudinal studies of high‐risk children for AN that include repeated measures of set‐shifting and central coherence.

### 4.1 Strengths and limitations

The present study has several strengths worth emphasising. To the best of our knowledge, this is the first study where daughters of women with a history of AN have been examined regarding cognitive functions. In the past, research on first‐degree relatives has mainly focused on adult sisters or mothers of women with AN. Targeting young children, we managed to investigate possible neuropsychological risk factors for AN before the expected age of ED onset. Regardless of our findings, greater knowledge about premorbid status in high‐risk groups for AN is much needed to detect early signs and facilitate adequate treatment interventions. Further, we have recruited an age‐matched comparison group to enable comparisons between girls at low risk of EDs. The majority of participants have been examined through extensive neuropsychological evaluations using well‐established tests. Apart from administering a full assessment of general intelligence, we relied on the most commonly used set‐shifting and central coherence tasks in ED research, including two different tests for measuring set‐shifting and two indices to assess central coherence. Systematic reviews of neuropsychological deficiencies in EDs have suggested that consistent use of instruments and outcome measures is crucial for comparing study results (Lang, Stahl, et al., 2014; Wu et al., 2014).

The limitations of this study are equally important to acknowledge. For comparative reasons, we decided to use test instruments commonly used in previous ED research. Even though the instruments are well‐established and considered appropriate for use in younger children, they have originally and generally been developed for an adult population. Further, the study was confined to a cross‐sectional design. The detection of possible subtle deviances in our sample may have been obstructed by examining neuropsychological functioning during an age characterised by significant cognitive development. Applying a longitudinal approach might have enabled less ambiguous interpretation of the results. For this reason, a 5‐year follow‐up study of our sample is intended. Ideally, future research should also explore the possible correlations of neuropsychological performance between mothers with AN and their unaffected daughters. Additional unexplored correlates relate to the known associations between neuropsychological functions and neuropsychiatric traits in AN. A subsequent publication will focus on the psychiatric morbidity including traits of autism and ADHD, and possible associations with neuropsychological functioning.

Another limitation of this study is the relatively small sample size, which could weaken the power and lead to failure to detect relevant differences between the groups. The original aim was to include 50 girls in each group. Despite quite an extensive period of recruitment, both in and outside of the Region Västra Götaland, we struggled to reach enough potential study participants. Another study complication was caused by the COVID pandemic, resulting in multiple cancellations and rescheduling of examinations, ultimately delaying and prolonging the recruitment process. Further, the high‐risk group was comprised of daughters of mothers with either current or previous AN. Combining the groups could be considered a possible limitation. Nevertheless, female offspring constitute a high‐risk group for AN development regardless of the mother's disease status. Finally, our study was confined to include female offspring of mothers with AN. The exclusion of male offspring could be considered another study limitation. Our decision was based on examining the first‐degree relatives at the highest risk of AN development, consequently selecting only females. Future research could investigate the premorbid function of sons of mothers with AN.

## 5 CONCLUSIONS

Contrary to the study hypotheses, our results did not confirm the presence of neuropsychological deviations regarding set‐shifting and central coherence abilities among girls at high risk of developing AN. An alternative interpretation is that the non‐significant findings may reflect the difficulty of measuring (subtle) neuropsychological inefficiencies in young children during the ages of peak cognitive development. A follow‐up study of our sample could elucidate further whether girls at high risk of AN display a deviant cognitive developmental trajectory compared with their low‐risk counterparts.

## CONFLICT OF INTEREST STATEMENT

The authors have no conflict of interest to report in this study.

## Data Availability

The data that support the findings of this study are available on request from the corresponding author. The data are not publicly available due to privacy or ethical restrictions.
